# Coping behaviour of female teachers: Demographic determinants

**DOI:** 10.4103/0972-6748.57856

**Published:** 2009

**Authors:** M. Chaturvedi, T. Purushothaman

**Affiliations:** Scientist-E, Scientist-B, D.R.D.O, Selection Centre Central, S.I. Lines, Bhopal, India

**Keywords:** Demographic variables, Stress-coping, Teachers

## Abstract

**Background:**

The study investigates the role of certain demographic variables in determining stress-coping behavior of female teachers.

**Materials and Methods:**

The sample consists of 150 female teachers selected by stratified sampling method from various schools of Bhopal. Stress-coping behavior was measured with the help of a subscale of ‘The Occupational Stress Indicator’ (Wendy Lord, 1993) consisting of 28 items encompassing six dimensions of coping strategies i.e. Logics, Involvement, Social Support, Task Strategies, Time Management and Home and Work Relations. The scores of the subjects were compared in terms of marital status, age, and level of teaching with the help of ‘*t*’ test and ‘F’ test was used for comparing experience.

**Results:**

Marital status, age, and experience were found to be significant determinants of stress-coping, whereas the sores did not differ significantly on the basis of level of teaching.

**Conclusion:**

Married teachers in the age range of 40-60 years, with higher experience can cope better with the job stress than their counterparts.

An individual cannot remain in a state of stress and strain and tries to adopt some strategy to deal with the stress. Stress is also defined as a mismatch between demands and coping resources. Coping refers to the thoughts and acts people use to meet the internal and external demands.

Coping has been defined as the behavioral and cognitive efforts a person uses to manage the demands of a stressful situation (Chang & Strunk, 1999). Folkman, Lazarus, Gruen, and DeLongis (1986) define coping as “the person’s cognitive and behavioral efforts to manage the internal and external demands in the person-environment transaction”. In times of stress, an individual normally engages in certain coping strategies to handle the stressful situations and their associated emotions. The more an individual adopts adaptive coping strategies, the less his/her stress, and better his/her mental health. There are several methods of coping. Feeling in Control as a Way of Coping (Rubin, Paplau, & Salovey, 1993), Optimism and Pessimism Coping Style (Rubin, Paplau, & Salovey, 1993), Approach and Avoidant Coping (Chang & Strunk, 1999), Appraisal and Coping (Rubin, Paplau, & Salovey, 1993). Endler & Purker (1990) gave three different coping styles i.e. task- oriented (problem-focused), emotion-oriented coping and avoidance-oriented coping.

Coping resources enable the individual to handle stressors more effectively, reduce the intensity of symptoms and help recover faster from exposure. These are adaptive capacities that provide immunity against damage from stress (Baum & Singer, 1982). These resources are psychological prophylaxis that can reduce likelihood of stress (Baum & Singer, 1982). Some studies have demonstrated a relationship between coping and mental health. Ebata and Moos (1994), Simoni and Peterson (1997) and Srivastava (1991) found that positive coping (e.g. problem solving action, logical analysis, information seeking) was positively related to wellbeing. In contrast, avoidance coping (e.g. denial or suppression of feelings) was associated with maladjustment to life stress. Kucukalic *et al*., (2003) emphasized that coping is a dynamic process that is reciprocally related between the individual and his environment. He found that the subjects who faced torture used more maladaptive coping than those who had not faced torture.

Evidence from the research efforts on science teacher stress suggests strategies such as meditation and relaxation and engagement in leisure-time activities for palliating stress (Betkouski, 1981; Penny, 1982). Trendall (1989) in his study on primary, secondary and special school teachers studied the relationship of age, sex, experience, qualification and level of responsibility with stress, strain and coping. Significant differences were found for sex group and level of qualification. It was found that the teachers with 5-10 years of experience experienced more stress, but senior teachers reported lesser stress.

In this study, coping was referred to as the cognitive and behavioral efforts used by an individual to handle difficulties and stress at work.

The aim of the present study was to investigate the influence of certain demographic factors in determining the stress-coping of female teachers.

## MATERIALS AND METHODS

The study was conducted on 150 female teachers from various schools of Bhopal. The stratified random sampling method was used for selection of sample. The age range varied from 20-60 years and the experience also varied from 1-35 years.

### Tool

The subscale of ‘The Occupational Stress Indicator’ (Wendy Lord, 1993) was used to assess stress-coping behaviour of the teachers. It is one of the subscales of the Battery consisting of 28 items comprising six dimensions of coping strategies i.e. Logics, Involvement, Social Support, Task Strategies, Time Management and Home and Work Relations. The subjects were required to rate themselves on a 6-point rating scale. Hence the range of scores on the test could be between 28 to168. The scores of the subjects were compared in terms of marital status, age, and level of teaching with the help of ‘*t*’ test and ‘F’ test was used for comparing experience.

## RESULTS AND DISCUSSION

Comparison of scores on the basis of marital status revealed significantly higher scores of married teachers on five dimensions of coping i.e. logics, social support, task strategies, time management, home and work relations as well as total score, indicating better coping ability of married teachers [Table 1]. Stress-coping is closely related to the overall life satisfaction of the individual (Baum and Singer, 1982). The status of marriage brings considerable satisfaction to both men and women but delivers special bonus to women in Indian society. Married women are not only happier than single women but they are also safer (Pant Amrita *et al*., 2002). On the other hand studies reveal that an overall satisfaction with life and workplace is much lower among unmarried women (Pant Amrita *et al*., 2002; Wadud *et al*., 1998). Better coping of their job stress by married women can be explained with ‘Spillover Model’ (Crouter A., 1984), which suggests that the emotional states experienced in one sphere get transferred to the other areas of life.

**Table 1:** Coping skills of married and unmarried teachers

Coping dimensions	Married N = 56		Unmarried N = 94		‘*t*’
	Mean	SD	Mean	SD	
Logics	25.21	2.06	20.72	4.24	3.74**
Involvement	20.62	4.36	18.17	4.77	2.66**
Social support	25.03	2.79	21.06	4.35	2.51*
Task strategies	24.45	3.36	20.45	4.33	2.58*
Time management	23.48	3.78	19.07	4.28	2.73**
Home and work relations	12.41	3.36	9.62	4.12	1.19
Total score	131.21	16.48	109.10	22.52	2.80**

* *P* < .05;

** *P* < .01

Age was found to positively affect the stress-coping scores. Women in the age range of 40-60 years scored significantly higher than the women in the younger age range on all the dimensions of coping i.e. logics, involvement, social support, task strategies, time management, home and work relations as well as total score [Table 2].

**Table 2:** Coping skills of teachers of different age groups

Coping dimensions	20-40 yrs, = 88		40-60 yrs, = 62		‘*t*’
	Mean	SD	Mean	SD	
Logics	22.08	3.54	24.82	2.71	3.26**
Involvement	18.26	3.89	21.96	4.11	5.60**
Social support	22.52	4.02	24.43	3.58	3.00**
Task strategies	22.15	4.12	24.00	3.78	2.80**
Time management	20.61	4.73	23.36	4.12	3.61**
Home and work relations	10.41	3.5	12.42	3.68	3.41**
Total score	105.63	22.42	129.73	19.56	2.64**

* *P* < .05;

** *P* < .01

Studies in the past also indicated that as the workers grow older they tend to cope better with their jobs (Glenn, Taylor and Weaver, 1977; Singh, 1980; Near *et al*., 1978, Wadud *et al*., 1998).

The findings on teaching experience indicated that the teachers with up to five years of experience scored much lesser on stress-coping than the teachers with more than five years of experience on all the dimensions of coping i.e. logics, involvement, social support, task strategies, time management, home and work relations as well as total score indicating that with increased experience the women are in a better position to cope with the job stress [Table 3] (Wadud *et al*., 1998). Trendall (1989) in his study on primary, secondary and special school teachers found that more stress was experienced by the teachers with 5-10 years of experience, but senior teachers reported lesser stress. Prakash *et al*., (2002) in their study of University teachers found no major differences between male and female teachers at varying teaching experience levels on measures of occupational role stressors and coping.

**Table 3:** Coping skills of teachers with different experience groups

Coping dimensions	1-5 yrs, N = 66		6-10 yrs, N = 34		10 yrs 1, N = 50		F
	Mean	S.D	Mean	S.D.	Mean	S.D.	
Logics	20.90	3.73	25.31	2.14	24.7	2.99	48.07***
Involvement	17.57	3.37	20.93	5.43	21.7	4.47	17.69***
Social support	21.52	4.38	25.25	2.59	25.15	2.82	33.61***
Task strategies	21.12	4.23	24.93	3.44	24.5	3.55	27.79***
Time management	19.60	4.47	23.56	4.12	23.75	4.10	25.26***
Home and work relations	9.75	3.43	12.56	3.36	12.85	3.56	17.54***
Total score	109.46	24.03	130.72	20.14	129.97	21.19	28.46***

* *P* < .05;

** *P* < .01

*** *P* < .001

The comparison of primary and high school teachers did not show a significant difference in terms of stress-coping scores except on the dimension of logics, in which the high school teachers scored higher than the primary school teachers [Table 4].

**Table 4:** Coping skills of primary and high school teachers

Coping dimensions	Primary teachers N = 78		High school teachers N = 72		‘*t*’
	Mean	SD	Mean	SD	
Logics	22.30	4.47	23.96	1.83	2.94**
Involvement	20.02	4.90	18.93	3.52	1.56
Social support	23.07	4.62	23.90	2.67	1.37
Task strategies	23.02	4.69	22.93	3.26	1.35
Time management	21.43	5.16	22.11	4.11	.878
Home and work relations	11.41	4.05	11.16	3.15	.42
Total score	112.76	27.42	110.81	18.67	1.78

** *P* < .01

Empirical research has generated some hard data to suggest the sensitivity of marital status, age and experience and level of teaching as some of the significant demographic variables in determining the coping with occupational stress of female teachers. These findings have gained added significance in view of the increasing influx of women into the workforce and consequent transition of traditional roles as women in the society.
